# The importance of provocation tests with COX2 inhibitors on patients with a history of cross-hypersensitivity to non-steroidal anti-inflammatory (NSAIDs)

**DOI:** 10.1186/1939-4551-8-S1-A213

**Published:** 2015-04-08

**Authors:** Tania Tavares Gonçalves

**Affiliations:** 1 Policlinica Geral Do Rio De Janeiro, Brazil

## Background

To verify the importance of provocation tests with COX2 selective inhibitors before release as an alternative medication, on patients with a record of cross-hypersensitivity to non-steroidal anti-inflammatory (NSAIDs)

## Methods

Retrospective analysis of patient records submitted to provocation tests with COX2 selected inhibitors, in Policlínica Geral do Rio de Janeiro, in the period between October 2010 and July 2014. The provocation tests were simple blind controlled placebo.

## Results

One hundred and twenty provocation tests were done with COX2 selected inhibitors, out of which, 116 with etoricoxib (96,6%) and 4 with celecoxib (3,3%). One hundred and two patients were female, with predominance in ages between 40 and 64 years old (41,8% of the women). Men represented only 15.9% of patients, with predominance in ages between 12 and 30 years old (40,10% of the men). Five positive tests occurred (4,1%), all of them tested with etoricoxib. In positive cases, 4 patients were female and 1 male, with predominance in ages between 17 and 29 years old, and 1 patient of 60 years old.

## Conclusions

The liberation of alternative medication for patients with cross-hypersensitivity to NSAIDs should be permitted only after the realization of provocation tests with COX2 selective inhibitors, given that 4% of the tests are positive.

